# A Longitudinal Study on Social Competence Development and Sleeping Habits

**DOI:** 10.2188/jea.JE20090148

**Published:** 2010-03-05

**Authors:** Etsuko Tomisaki, Emiko Tanaka, Ryoji Shinohara, Yuka Sugisawa, Lian Tong, Maki Hirano, Taeko Watanabe, Yoko Onda, Yukiko Mochizuki, Yuri Kawashima, Yuko Yato, Noriko Yamakawa, Tokie Anme, Japan Children’s Study Group

**Affiliations:** 1 Research Institute of Science and Technology for Society, Japan Science and Technology Agency, Tokyo, Japan; 2 Graduate School of Comprehensive Human Sciences, University of Tsukuba, Tsukkuba, Ibaraki, Japan; 3 College of Letters, Ritsumeikan University, Kyoto, Japan; 4 Clinical Research Institute, Mie-Chuo Medical Center National Hospital Organization, Tsu, Japan

**Keywords:** social competence, sleeping habit, longitudinal study

## Abstract

**Background:**

It is known that sleep problems impact children’s health, learning, and school performance. The purpose of this paper is to examine the association between sleeping habits and social competence development.

**Methods:**

Three hundred and nine caregiver-child dyads participated in this study, which was conducted as part of a Japan Science and Technology Agency (JST) project. The caregivers answered some questionnaires about sleeping habits when the child was 9 months and 18 months old. Caregiver-child interaction was observed when the child was 30 months old, and the features of the interaction were examined using the Interaction Rating Scale (IRS) as a measure of social competence.

**Results:**

The caregivers’ attitude toward sleeping in the 9-month period was found to be significantly correlated with the children’s social competence at 30 months. Moreover the caregivers’ attitude toward sleeping in the 9-month period significantly correlated with the children’s sleeping habits at 9 and 18 months.

**Conclusions:**

These findings show that the caregivers’ attitude toward sleeping is an important factor influencing the development of children’s social competence.

## INTRODUCTION

It is universally accepted that sleep problems impact children’s health, learning, and school performance. Inadequate sleep can adversely affect all aspects of a child’s biopsychosocial health.^1^ Studies have focused on children’s sleep in relation with obesity^2^^–^^4^ and behavioral problems.^5^^–^^8^ Gregory^5^ reported that sleep problems may forecast behavioral/emotional problems. Further Dahl^6^ reported that inadequate sleep results in tiredness, difficulties with focused attention, low threshold to express negative affect (irritability and easy frustration), and difficulty in modulating impulses and emotions.

Kohyama et al^9^ reported 3-year-olds in Japan sleep particularly late as compared to those in other countries. In 2000 Kawai^10^ reported that 52% of 3-year-olds in Japan went to bed later than 10:00 pm. Kohyama et al^11^ reported that 43.0% of 307 18-month-olds, and 53.7% of 151 36-month-olds living in Tokyo had a sleep onset time of 10:00 pm or later. Later bedtimes are specifically associated with shorter nocturnal sleep durations.^9^^,^^12^ Touchette^7^ reported that children with short nocturnal sleep durations before the age of 3.5 years show an increased risk of high hyperactivity–impulsivity and low cognitive performance at 6 years as compared to children who sleep 11 hours each night. According to a preschool children report by Yokomaku et al,^8^ late risers, late sleepers, irregular risers, and irregular sleepers were likely to exhibit problematic behaviors.

Studies have examined the associations between sleeping habits, sleep duration, and behaviors, but not many have examined the social development in terms of (1) autonomy, (2) responsiveness, (3) empathy, (4) motor regulation, and (5) emotional self-regulation. The present study analyzes how parent’s attitude toward sleeping in 9 months correlates with the child’s social development at 30 months.

## METHODS

### Participants

The participants comprised 309 caregiver-child dyads, who participated in the Japan Science and Technology Agency (JST) project. The participants were recruited from two Japanese cities (Oosaka, Mie). The 309 dyads were observed at 30 months of the children’s development. The caregivers completed a questionnaire regarding their attitudes toward sleeping and their children’s sleeping habits at 9 and 18 months since the children’s birth, and demographic data at 9 months.

In order to comply with the ethical standards laid down by the JST, before conducting the research, the families of all the participants signed informed consent forms and were made aware that they had the right to withdraw from the experiment at any time. As the infants were too young to provide informed consent, we carefully explained the purpose, content, and methods of the study to the caregivers and obtained their consent. To maintain the confidentiality of the participants their personal information was collected anonymously and stored securely using a personal ID system. Further, all the image data were stored on a password-protected disk, only the researchers who were granted permission by the chairman were given access to the data. This study was approved by the ethics committee of the JST.

### Materials

The Interaction Rating Scale (IRS) was used in a controlled laboratory environment to rate the children’s social competence on the basis of the observation of the caregiver-child interaction. The reliability and validity of this tool has been already examined.^13^^–^^15^ The IRS includes 70 items for a behavioral score and 11 items for an impression score, which were grouped into 10 subscales. Five subscales focus on children’s social competences: (1) Autonomy, (2) Responsiveness, (3) Empathy, (4) Motor regulation, and (5) Emotional regulation. Another five subscales assess the caregiver’s parenting skills: (6) Respect for autonomy development, (7) Respect for responsiveness development, (8) Respect for empathy development, (9) Respect for cognitive development, and (10) Respect for socioemotional development. One item assesses the overall impression of synchronous relationships. Each subscale assesses the presence of behavior (1 = Yes, 0 = No), and the sum of all the items in the subscale provides the overall behavior score.

### Procedure

A questionnaire survey and participant observation were carried out. The primary caregivers (mostly mothers) filled in the questionnaire on the caregivers’ general child rearing practices and seeking information about the children at 9 and 18 months, including the children’s gender, family type, sibling, mothers’ and fathers’ age, mothers’ and fathers’ career, and family’s annual income (Table 1).

**Table 1.** Demographic Information

Items	*n*	%
Gender		
Boys	150	48.5
Girls	159	51.5
Family type		
Nuclear family	272	88.0
Extended family	31	10.0
No answer	6	1.9
Siblings		
No	170	55.0
Yes	137	44.3
No answer	2	0.6
Mother’s career		
No	165	53.4
Yes	144	46.6
Mother’s education		
Middle school	9	2.9
High school	64	20.7
Vocational school	71	23.0
Short-term college education	82	26.5
University	76	24.6
Post-college education	3	1.0
No answer	4	1.3
Father’s career		
No	0	0.0
Yes	309	100.0
Father’s education		
Middle school	5	1.6
High school	96	31.1
Vocational school	45	14.6
Short-term college education	6	1.9
University	120	38.8
Post-college education	20	6.5
No answer	17	5.5
Family’s annual income		
Less than JPY 2 million	15	4.9
JPY 2–4 million	83	26.9
JPY 4–6 million	136	44.0
JPY 6–8 million	40	12.9
JPY 8–10 million	16	5.2
More than JPY 10 million	13	4.2
No answer	6	1.9
Mother’s age		
20–29	95	30.7
30–39	201	65.0
40–49	12	3.9
No answer	1	0.3
Father’s age		
20–29	72	23.3
30–39	196	63.4
40–49	23	7.4
50–	4	1.3
No answer	14	4.5

JPY: Japanese yen.

In the participant observation stage, we videotaped the caregiver-child interactions in a room having five video cameras, one at each of the four corners of the room and one at the centre of the ceiling. These recordings were made at 30 months since the children’s birth. The dyads were escorted to a playroom (4 × 4 m) furnished with a small table and a chair meant for children. Each caregiver was asked to teach his/her child to carry out a prescribed task, which would have been slightly difficult for the child to accomplish by himself/herself. (In this study, the task involved building a small house with three building blocks.) During the process, the caregiver gave the necessary instructions and helped the child as he/she does in daily life. The task was considered to begin from the time the caregiver obtained the building blocks from the research staff and was considered to finish upon the completion of the task and tidying up of the play area by the caregiver. The observation period typically lasted for 1–5 min.

An evaluator completed the IRS checklist composed of 25 items focusing on children’s behavior toward the caregivers (eg, child looks at the caregiver’s face as a social reference) and 45 items focusing on the caregiver’s behavior. The total score for each child was the sum of the score that he/she received on all the subscales. A higher score indicated a higher level of development.

### Analysis

The Statistical Analysis System (SAS, ver. 9.1) was used for the analysis. The questionnaire on sleeping comprised question on the “Regular sleep-wake rhythm” and “Sleeping habits” where the rating were 1 = very important, 2 = important, 3 = not very important, 4 = not important. The rating for “Mother’s sleeping time and wake up time” were 1 = almost constant, 2 = little irregular, 3 = very irregular. The last three items were “The time the child wants to have a nap differs everyday by more than 1 hour.” “Child feels sleepy at almost the same time,” and “The time at which the child wakes up differs everyday by more than 1 hour.” The rating for these items were 1 = not true most of the time, 2 = rarely true, 3 = more likely that it is not true, 4 = more likely that it is true, 5 = rarely not true, 6 = true most of the time. We assessed the distribution of the rating to these items between the children’s social competence.

## RESULTS

There was no relationship between the children’s social competence and their demographic data at 9 months. The children whose caregivers did not give much importance to living habits showed significantly lower social competence scores in every subscales (autonomy: *r* = 0.121 *P* = 0.034, responsiveness: *r* = 0.149 *P* = 0.009, empathy: *r* = 0.136 *P* = 0.017, motor regulation: *r* = 0.140 *P* = 0.014, emotional regulation: *r* = 0.112 *P* = 0.049) (Table 2). Children who did not have a regular sleeping habit in 18 months showed significantly lower social competence scores in autonomy (*r* = 0.118 *P* = 0.039) and empathy (*r* = 0.128 *P* = 0.024) (Table 3).

**Table 2.** The relationship between mother’s attitude toward sleeping and child’s social competence

	Items	30 months											
	
		Social competence	*P*	Autonomy	*P*	Responsiveness	*P*	Empathy	*P*	Motor regulation	*P*	Emotional regulation	*P*
9 months	Maintaning sleep-wake rhythm	0.175	0.002	0.121	0.034	0.149	0.009	0.136	0.017	0.140	0.014	0.112	0.049
	sleeping habit	0.128	0.024	0.076	n.s	0.164	0.004	0.096	n.s	0.114	0.045	0.058	n.s
	mother’s sleeping habit	0.006	n.s	0.130	0.022	0.078	n.s	−0.013	n.s	−0.059	n.s	−0.064	n.s

**Table 3.** The relationship between child’s sleeping habit and child’s social competence

	Items	Autonomy	*P*	Responsiveness	*P*	Empathy	*P*	Motor regulation	*P*	Emotional regulation	*P*
18 months	The child’s wake up time changes everyday by more than 1 hour	0.118	0.039	0.100	n.s	0.128	0.024	−0.033	n.s	0.045	n.s

There was correlation between the caregiver’s attitude toward sleeping and the child’s sleeping habit in 9 months (Naptime differs everyday by more than 1 hour: *r* = 0.113 *P* = 0.047, Child’s wake up time changes everyday by more than 1 hour: *r* = 0.138 *P* = 0.015) and 18 months (Child’s wake up time changes everyday by more than 1 hour: *r* = 0.155 *P* = 0.007) (Table 4).

**Table 4.** The relationship between mother’s attitude toward sleeping and child’s sleeping habit

	Items	9 months						18 months	
		
		Naptime differs everyday by more than 1 hour	*P*	Child feels sleepy at almost the same time	*P*	Child’s wake up time changes everyday by more than 1 hour	*P*	Child’s wake up time changes everyday by more than 1 hour	*P*
9 months	Maintaining sleep-wake rhythm	0.113	0.047	0.101	n.s	0.138	0.015	0.155	0.007
	Sleeping habit	0.032	n.s	0.019	n.s	0.022	n.s	0.173	0.002
	Mother’s sleeping habit	0.177	0.002	0.135	0.018	0.237	<0.0001	0.259	<0.0001

## DISCUSSION

The feature of this study is the following point.

First, this study examined more than 300 dyads, and they all associated in the test when their children were at 9, 18 and 30 months.

Secondly, all of the participants’ social competence had been examined by using the IRS.

Third, the caregivers’ attitude toward sleeping when children were 9 and 18 months old was a very important factor influencing the children’s social competence at 30 months. The children whose caregivers did not give importance to sleeping did not have a regular sleeping habit in 9 and 18 months. Moreover, the children who did not have a regular sleeping habit in 9 and 18 months had low social competence scores in terms of autonomy, emotional regulation and empathy. Not many studies have investigated this aspect, and exploring it further might reveal valuable information.

Although this study reports on a new aspect in caregiver-child dyads, it has certain limitations. First, we examined children only when they were 9, 18 and 30 months old. Second, the questionnaire was administered only to the children’s caregivers. Third, we did not seek the exact time of the sleeping habits such as waking up time, naptime, and sleeping time.

We are currently in the process of analyzing the data collected over a period of 42 months. In this examination, there are validity in the questionnaire about sleeping. We hope to obtain much more interesting results with regard to the relationship between social development and sleeping habits in this follow-up study.
